# Uso de Índices Aterogênicos como Métodos de Avaliação das Doenças Ateroscleróticas Clínicas

**DOI:** 10.36660/abc.20230418

**Published:** 2023-12-15

**Authors:** Yuri Barbosa Araújo, Ana Beatriz Rocha Almeida, Márcio Fellipe Menezes Viana, Rafael Alexandre Meneguz-Moreno

**Affiliations:** 1 Universidade Federal de Sergipe Departamento de Medicina de Lagarto Lagarto SE Brasil Universidade Federal de Sergipe - Departamento de Medicina de Lagarto, Lagarto, SE – Brasil

**Keywords:** Aterosclerose, Placa Aterosclerótica, Lipoproteínas, Índice de Perfusão

## Abstract

**Fundamento:**

A busca por métodos clinicamente úteis de avaliação de doenças ateroscleróticas, com boa acurácia, de baixo custo, sem invasividade e de fácil manejo, há anos vem sendo estimulada. Dessa forma, os índices aterogênicos avaliados deste estudo podem se encaixar nesta demanda crescente.

**Objetivos:**

Avaliar o potencial dos índices aterogênicos como métodos de avaliação de pacientes portadores de aterosclerose clínica.

**Métodos:**

Estudo transversal de centro único, por meio do qual foram avaliados os índices de Castelli I e II, índice aterogênico plasmático (IAP), índice de combinação de lipoproteínas e a variação do índice de perfusão periférica entre 90 e 120 segundos após um estímulo vasodilatador endotélio-dependente (ΔIPP_90-120_) na predição de aterosclerose. A significância estatística foi estabelecida em p < 0,05.

**Resultados:**

A amostra foi composta por 298 indivíduos com idade média de 63,0 ± 16,1 anos, dos quais 57,4% eram mulheres. Comparações pareadas da análise curva ROC dos índices que alcançaram área sob a curva (ASC) > 0,6 mostram que ΔIPP_90-120_ e IAP foram superiores aos demais índices, sem diferenças observadas entre si (diferença entre ASC = 0,056; IC95% -0,003-0,115). Ademais, tanto a ΔIPP_90-120_ [odds ratio (OR) 9,58; IC95% 4,71-19,46] quanto o IAP (OR 5,35; IC95% 2,30-12,45) foram preditores independentes de aterosclerose clínica.

**Conclusões:**

O IAP e ΔIPP_90-120_ apresentaram melhor acurácia para discriminar aterosclerose clínica. Além disso, foram preditores independentes de aterosclerose clínica, evidenciando uma possibilidade promissora para o desenvolvimento de estratégias preventivas e de controle para doenças cardiovasculares. Tratam-se, portanto, de marcadores adequados para estudos multicêntricos do ponto de vista de praticidade, custo e validade externa.

## Graphical abstract

**Figura Central:**
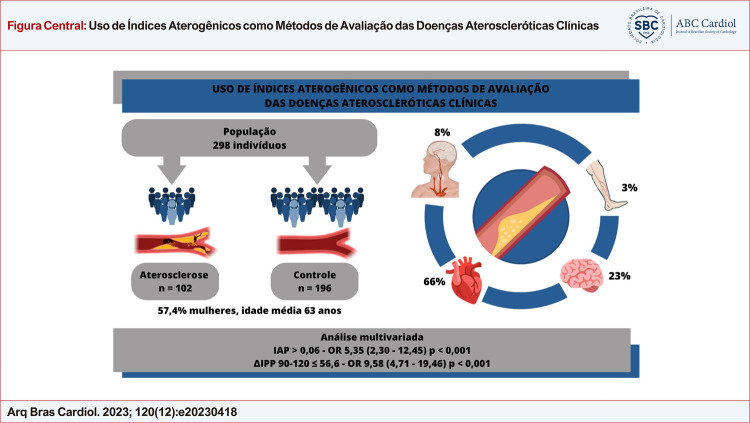
: Uso de Índices Aterogênicos como Métodos de Avaliação das Doenças Ateroscleróticas Clínicas

## Introdução

A aterosclerose é o pilar central da fisiopatologia de diversas doenças cardiovasculares.^1^ Apesar do uso difundido dos parâmetros lipídicos clássicos, amplamente disponíveis para análise clínica, outros parâmetros vêm sendo discutidos atualmente, propondo associações destas variáveis lipídicas com o intuito de avaliar as relações entre elas e a correlação com desfechos clínicos, especialmente a doença coronariana.^2-4^

Em 1983, Castelli sugeriu os Índices de Castelli I e II, como reflexo da depuração do colesterol total (CT) e do LDL, ambas mediadas pelos níveis de HDL.^5^ Recentemente, o índice aterogênico plasmático (IAP) tem ganhado notoriedade científica. Especula-se que o grande potencial preditivo do IAP para as doenças ateroscleróticas deriva da capacidade deste índice de indicar que a relação entre os triglicerídeos (TG) e o HDL pode predeterminar a direção preferencial do transporte intravascular de colesterol em direção a HDL benéficos ou LDL aterogênicos.^6,7^ Nos últimos 4 anos, representado pela relação entre as concentrações molares do CT, LDL e TG com o HDL, o índice de combinação de lipoproteínas (ICL) foi proposto como um possível preditor independente de doença coronariana em mulheres menopausadas.^8^

Recentemente, um estudo mostrou resultados animadores do índice de perfusão periférica (IPP), um parâmetro derivado do oxímetro de pulso, na avaliação da função endotelial na presença de aterosclerose. Este mesmo estudo relatou que o intervalo da variação do IPP entre 90 e 120 segundos (ΔIPP_90-120_) após a hiperemia reativa parece ter a maior correlação entre fatores de risco cardiovascular e disfunção endotelial.^9^

Dada a importância da disfunção endotelial e do perfil lipídico para o desenvolvimento e progressão das doenças ateroscleróticas, a busca por métodos de avaliação clinicamente úteis, com boa acurácia, de baixo custo, sem invasividade e de fácil manejo há anos vem sendo estimulada. Tendo em vista a já descrita associação destes índices com diversos desfechos clínicos cardiovasculares,^3,4,7,10-19^ eles podem se encaixar na demanda crescente de custo-efetividade e os tornam atraentes para futuros ensaios e possível melhoria na detecção, prevenção e tratamento de tais doenças. Logo, o presente estudo teve como objetivo avaliar o potencial dos índices aterogênicos como métodos de predição de doença aterosclerótica clínica.

## Métodos

### Delineamento do estudo

Este é um estudo observacional, do tipo transversal, por meio do qual foram avaliados os valores do índices de Castelli I e II, índice aterogênico plasmático, índice de combinação de lipoproteínas e a variação do índice de perfusão periférica após um estímulo vasodilatador endotélio-dependente. Foram avaliados pacientes com aterosclerose clínica em diversos sítios vasculares, baseados na concomitância de sítios envolvidos e na sua característica sistêmica.^20^

### Local do estudo e cálculo amostral

O presente estudo foi realizado em ambulatórios de cardiologia, endocrinologia e geriatria vinculados a um hospital terciário em uma cidade do nordeste brasileiro. Foi realizado um cálculo retrospectivo do tamanho da amostra para o desfecho primário com uma proporção para ocorrência do desfecho de 1:2, baseado em estudo piloto previamente realizado. Com um poder de 0,8, um α de 0,05 e uma ASC=0,6 conforme nossa hipótese a-priori (hipótese nula: ASC=0,5), foi necessária uma amostra de 294 participantes. Para compensar possíveis perdas, foi acrescido 10% para ajuste da amostra, totalizando 323 participantes.

### Critérios de inclusão e exclusão

Todos os pacientes que comparecessem ao atendimento ambulatorial das especialidades supracitadas seriam convidados a participar do estudo desde que tivessem pelo menos 18 anos e um resultado de lipidograma coletado até 03 meses antes da inserção na pesquisa. Em razão a alterações particulares dos parâmetros lipídicos, os pacientes com hipercolesterolemia familiar e usuários de inibidores da protease, anticoncepcionais orais combinados, ou isotretinoína foram excluídos do estudo. Ademais, uma vez que inúmeros fatores podem afetar a reatividade vascular, pacientes renais dialíticos, gestantes e pacientes que tenham se exercitado 1 hora antes da entrevista, ou que tenham ingerido substâncias energéticas, ou que tenham fumado ao menos 4 a 6 horas antes do início da coleta também foram excluídos do estudo.

### Definição de doença aterosclerótica clínica

Os pacientes tiveram suas doenças confirmadas pelo prontuário eletrônico elaborado por médicos especialistas com auxílio de exames complementares, entre os quais: laudos de cineangiocoronariografia e angiotomografia de coronárias com placas ateroscleróticas com estenose ≥ 50%, ecocardiograma com estresse físico ou farmacológico, ressonância magnética cardíaca com estresse farmacológico, arteriografia ou ecodoppler arterial de membros inferiores e ecodoppler de carótidas evidenciando placas ateroscleróticas com estenose ≥ 50%, além de tomografia e angiotomografia de crânio com sinais de isquemia e excluídas etiologias cardioembólicas. As provas não-invasivas foram consideradas positivas quando fosse evidenciada isquemia. Foram considerados laudos positivos de aterosclerose diagnosticada até 1 ano do lipidograma mais recente.

### Alocação dos grupos

Neste estudo, o grupo aterosclerose clínica foi composto por: doença arterial coronariana, doença aterosclerótica carotídea ou periférica e doença cerebrovascular isquêmica aterotrombótica. Desta forma, o grupo controle foi composto por aqueles que não tinham doença aterosclerótica clínica diagnosticada, isto é, indivíduos com aterosclerose subclínica ou sem processo de aterosclerose.

### Coleta de dados

A coleta de dados foi realizada no período de janeiro de 2022 a dezembro de 2022, por meio de entrevista e exame físico em salas individualizadas, com portas fechadas, respeitando a privacidade do participante e a lei geral de proteção de dados. Os entrevistados foram aleatoriamente selecionados por busca ativa em dias aleatórios, previamente ao atendimento ambulatorial.

Foram coletadas variáveis relacionadas ao risco cardiovascular: sexo, idade, etnia, prática regular de atividade física, índice de massa corporal (IMC), dislipidemias, diabetes mellitus tipo 2, hipertensão arterial sistêmica e história de etilismo e tabagismo atual ou prévio. Aqueles que não praticassem de forma regular ao menos 150 minutos de atividade física moderada foram classificados como prática inadequada de atividade física.^21^

Foram calculados os seguintes índices aterogênicos: índices de Castelli I (IC-I) (CT/HDL) e Castelli II (IC-II) (LDL/HDL), o índice de combinação de lipoproteínas (ICL) (CTxTGxLDL/HDL), o índice aterogênico plasmático (IAP), calculado como log_10_(TG/HDL), e a variação do Índice de Perfusão Periférica no intervalo 90-120 segundos (ΔIPP_90-120_) após a desinsuflação do manguito. Para os cálculos do IAP e do ICL, os parâmetros lipídicos (CT, LDL, HDL e TG) foram expressos em mmol/L.

### Coleta do IPP

Para análise do IPP, foi utilizado um oxímetro de pulso portátil (modelo HC261, Multilaser, Brasil). Nesta avaliação, realizada por um investigador único, os pacientes foram acomodados, sentados, por aproximadamente 5 minutos numa sala silenciosa com temperatura controlada a 20-22 ºC. O protocolo de coleta do IPP seguiu o mesmo utilizado por Menezes et al.^9^ A partir da desinsuflação do manguito, o valor do IPP foi avaliado e registrado 90 e 120 segundos para avaliação da sua variação do IPP neste período (ΔIPP_90-120_) por meio da seguinte fórmula:


△IPP: (IPP tempo - IPP basal) / IPP basal (x 100)

### Análise estatística

Variáveis com distribuição normal foram descritas como média ± desvio padrão e variáveis sem distribuição normal foram descritas como mediana e intervalo interquartil. As variáveis contínuas foram avaliadas pelo método analítico de Shapiro-Wilk para determinar a normalidade da distribuição. Foi realizado o teste t de Student não pareado para variáveis com distribuição normal e o teste U de Mann-Whitney para aquelas sem distribuição normal. Para as variáveis categóricas, foi utilizado o teste do qui-quadrado de Pearson. Os pontos de corte para os índices aterogênicos foram obtidos pela curva *receiver operating characteristic* (ROC), escolhidos por meio do índice de Youden. As áreas sob a curva (ASC) foram calculadas e comparadas pelo método DeLong. Ademais, foram registrados a sensibilidade, a especificidade, o valor preditivo positivo (VP+) e negativo (VP-) e as razões de verossimilhança positiva (RV+) e negativa (RV-) para ocorrência do desfecho.

A análise de correlação de Pearson foi realizada para investigar a correlação dos índices com maior ASC com outras variáveis contínuas. Para avaliar o grau de associação das variáveis ao desfecho, foram calculadas as *odds ratios* (OR) e seus intervalos de confiança de 95% (IC_95%_) para presença de doença aterosclerótica por meio da regressão logística univariada. Aquelas que alcançaram p<0,10 ou que fossem consideradas clinicamente relevantes foram incluídas no modelo multivariado. Valores de p<0,05 foram considerados estatisticamente significativos. Os dados foram analisados utilizando os *softwares* SPSS, versão 26.0 (SPSS Inc., Chicago, IL, EUA) e MedCalc®, versão 19.5 (MedCalc Software Ltd, Ostend, Belgium).

### Aspectos éticos

Este projeto foi aprovado pelo Comitê de Ética em Pesquisa, sob parecer nº 5.106.513, conforme diretrizes e normas estabelecidas na resolução nº 466/2012 do CNS, a qual versa sobre pesquisas com seres humanos.

## Resultados

No período da pesquisa, foram analisados dados de 323 voluntários, dos quais 13 foram excluídos por uso de anticoncepcionais e 4 por uso de inibidores da protease, 5 por serem pacientes dialíticos, 2 por diagnóstico de hipercolesterolemia familiar e 1 por ser gestante. Sendo assim, a amostra final foi composta por 298 participantes (idade média 63±16,1 anos), dos quais 102 compuseram o grupo aterosclerose clínica, enquanto 196 participantes sem aterosclerose ou com aterosclerose subclínica compuseram o grupo controle. Entre os pacientes do grupo aterosclerose, os leitos arteriais mais acometidos por aterosclerose clínica foram, respectivamente, o coronariano (76), seguido pelo encefálico (26), carotídeo (9) e periférico (4); 12 pacientes foram diagnosticados com mais de uma doença aterosclerótica. As características clínicas basais dos grupos estudados estão resumidas na Tabela 1.

**Tabela 1:** – Comparação das características clínicas da população estudada

	População geral (n = 298)	Grupo		p

		Controle (n = 196)	Aterosclerose (n = 102)	
Idade, (anos)	63,0 ± 16,1	59,4 ± 17,0	70,0 ± 11,4	<0,001
Sexo feminino, n (%)	171 (57,4)	123 (62,8)	48 (47,1)	0,009
Não brancos, n (%)	265 (88,9)	172 (87,8)	93 (91,2)	0,372
IMC, (kg/m^2^)	28,4 ± 5,8	28,3 ± 6,4	28,6 ± 4,6	0,342
PAS, (mmHg)	132,6 ± 19,3	132,1 ± 18,1	133,5 ± 21,5	0,947
PAD, (mmHg)	80,1 ± 12,6	81,8 ± 12,0	77,0 ± 13,2	0,002
Frequência cardíaca, (bpm)	75,5 ± 13,3	76,7 ± 12,8	73,3 ± 14,0	0,015
Diabetes mellitus tipo 2, n (%)	128 (43,0)	70 (35,7)	58 (56,9)	<0,001
Dislipidemias, n (%)	196 (65,8)	106 (54,1)	90 (88,2)	<0,001
Hipertensão Arterial, n (%)	239 (80,2)	144 (73,5)	95 (93,1)	0,002
Tabagismo, n (%)	131 (44,0)	70 (35,7)	61 (59,8)	<0,001
Etilismo, n (%)	106 (35,7)	64 (32,7)	42 (41,6)	0,128
Atividade física regular, n (%)	60 (20,1)	51 (26,0)	9 (8,8)	<0,001
BRA/Inibidores da ECA, n (%)	212 (71,1)	131 (66,8)	81 (79,4)	0,023
Diuréticos, n (%)	154 (51,7)	93 (47,4)	61 (59,8)	0,043
Betabloqueadores, n (%)	117 (39,3)	50 (25,5)	67 (65,7)	<0,001
BCC, n (%)	83 (27,9)	50 (25,5)	33 (32,4)	0,211
Estatinas, n (%)	187 (62,8)	92 (46,9)	95 (93,1)	<0,001
Antiagregantes, n (%)	104 (34,9)	19 (9,7)	85 (83,3)	<0,001

IMC: índice de massa corporal; PAS: pressão arterial sistólica; PAD: pressão arterial diastólica; ECA: enzima conversora da angiotensina; BRA: bloqueadores do receptor de angiotensina; BCC: bloqueadores dos canais de cálcio.

Os parâmetros laboratoriais e os índices aterogênicos são apresentados na Tabela 2. Entre os parâmetros lipídicos, foi observada diferença apenas entre os níveis de triglicérides e HDL. No grupo aterosclerose, foram observados níveis maiores de triglicérides e menores de HDL. Os índices aterogênicos IC-I, IC-II, IAP, ICL mostraram-se significativamente maiores no grupo aterosclerose, ao passo que foi observada menor mediana da ΔIPP_90-120_.

**Tabela 2:** – Comparação dos parâmetros laboratoriais dos grupos de estudo

	População geral (n = 298)	Grupo		p

		Controle (n = 196)	Aterosclerose (n = 102)	
Colesterol total, (mg/dL)	175 (143 – 215)	178 (150 – 214)	162 (136 – 220)	0,250
Triglicérides, (mg/dL)	118 (85 – 158)	104 (77 – 138)	147 (114 – 188)	<0,001
Colesterol HDL, (mg/dL)	47 (39 – 56)	51 (42 – 59)	40 (34 – 47)	<0,001
Colesterol LDL, (mg/dL)	101 (72 – 132)	103 (74 – 134)	95 (69 – 132)	0,361
Colesterol não HDL, (mg/dL)	127 (95 – 162)	128 (93 – 160)	127 (99 – 168)	0,446
Hemoglobina, (g/dL)	13,2 ± 1,7	13,1 ± 1,7	13,2 ± 1,7	0,984
Hematócrito, (%)	39,9 ± 5,1	39,5 ± 5,0	40,6 ± 5,3	0,176
Plaquetas, (10^3^/mL)	238 ± 72	236 ± 69	243 ± 75	0,902
Leucócitos, (10^3^/mL)	6,35 (5,20 – 7,80)	6,30 (4,98 – 7,70)	6,49 (5,50 – 7,85)	0,102
Ureia, (mg/dL)	34 (27 – 44)	32 (26 – 40)	38 (29 – 50)	0,002
TFGe, (mL/min/1,73m^2^)	77 ± 23	81 ± 23	70 ± 22	<0,001
HbA1c, (%)	6,0 (5,5 – 6,8)	5,8 (5,4 – 6,4)	6,4 (5,9 – 7,7)	<0,001
Glicemia de jejum, (mg/dL)	97 (86 – 118)	93 (85 – 110)	105 (91 – 134)	<0,001
Índice de Castelli I	3,6 (3,0 – 4,7)	3,4 (2,8 – 4,3)	4,0 (3,4 – 5,4)	<0,001
Índice de Castelli II	2,1 (1,5 – 2,9)	2,0 (1,4 – 2,8)	2,3 (1,8 – 3,3)	0,011
Índice Aterogênico Plasmático	0,03 (-0,12 – 0,20)	-0,06 (-0,20 – 0,11)	0,17 (0,08 – 0,36)	<0,001
ICL	11,6 (6,7 – 24,2)	10,5 (5,6 – 20,3)	15,6 (8,4 – 35,7)	<0,001
IPP basal, (%)	5,3 (3,1 – 8,0)	4,0 (2,7 – 6,1)	7,8 (5,5 – 9,8)	<0,001
ΔIPP_90-120_, (%)	75,8 (45,3 – 130,3)	99,3 (69,4 – 157,8)	40,3 (8,1 – 65,0)	<0,001

HDL: lipoproteína de alta densidade; LDL: lipoproteína de baixa densidade; TFGe: taxa de filtração glomerular estimada pela fórmula CKD-EPI; ICL: índice de combinação de lipoproteínas; IPP: índice de perfusão periférica; ΔIPP90-120: a variação do índice de perfusão periférica no intervalo 90-120 segundos após a desinsuflação do manguito.

**Figura 1:**
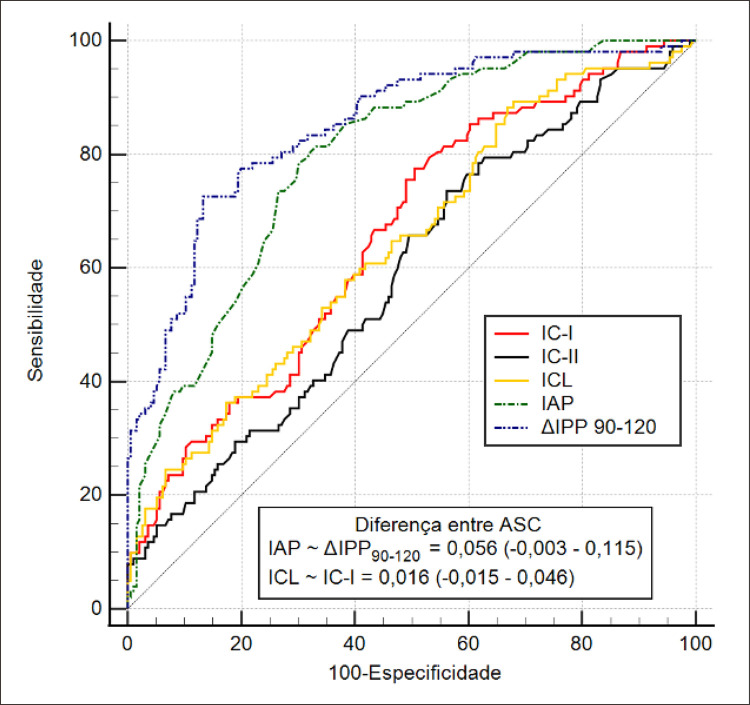
– Curvas ROC dos índices aterogênicos para doenças ateroscleróticas. IC-I: índice de Castelli I; IC-II: índice de Castelli II; ICL: índice de combinação de lipoproteínas; IAP: índice aterogênico plasmático; ΔIPP90-120: a variação do índice de perfusão periférica no intervalo 90-120 segundos após a desinsuflação do manguito.

A Tabela 3 apresenta os valores de sensibilidade, especificidade, valores preditivos e razões de verossimilhança dos índices analisados nesse estudo. Nota-se que apenas o Índice de Castelli II não alcançou uma ASC>0,6 (ASC=0,589). As curvas ROC destes índices podem ser observadas na Figura 1.

**Tabela 3:** – Sensibilidade, especificidade, valores preditivos e razões de verossimilhança dos índices aterogênicos

Índices	Ponto de corte	ASC	SEN	ESP	VPP	VPN	RVP	RVN
IC-I	>3,35	0,658	77,5	49,5	44,4	80,8	1,53	0,46
IC-II	>1,86	0,589	73,5	43,9	40,5	76,1	1,31	0,60
ICL	>6,90	0,642	88,2	33,2	40,7	84,4	1,32	0,35
IAP	>0,06	0,795	81,4	66,4	56,1	87,3	2,45	0,28
ΔIPP_90-120_	≤56,6	0,851	72,5	86,7	74,0	85,9	5,47	0,32

ASC: área sob a curva; SEN: sensibilidade; ESP: especificidade; VPP: valor preditivo positivo; VPN: valor preditivo negativo; RVP: razão de verossimilhança positiva; RVN: razão de verossimilhança negativa; IC-I: índice de Castelli I; IC-II: índice de Castelli II; IAP: índice aterogênico plasmático; ICL: índice de combinação de lipoproteínas; ΔIPP_90-120_: a variação do índice de perfusão periférica no intervalo 90-120 segundos após a desinsuflação do manguito.

Comparações pareadas da análise ROC dos índices que alcançaram ASC > 0,6 mostram que, embora não houvesse diferença significativa entre a ΔIPP_90-120_ e o IAP, ambos se mostraram superiores ao IC-I e ICL, entre os quais também não foi observada diferença.

Após observar a maior acurácia do IAP e ΔIPP_90-120_, foi realizada análise de correlação de Pearson para investigar suas correlações com outras variáveis contínuas. O IAP foi positivamente correlacionado com a idade (r=0,173, p=0,003), IMC (r=0,116, p=0,046), CT (r=0,138, p=0,017), TG (r=0,830, p<0,001), e foi negativamente correlacionado com o HDL (r=-0,599, p<0,001) e ΔIPP_90-120_ (r=-0,237, p<0,001). Por sua vez, a ΔIPP_90-120_ foi positivamente correlacionada com a PAD (r=0,154, p=0,012), HDL (r=0,321, p<0,001) e negativamente correlacionada com a idade (r=-0,258, p<0,001) e TG (r=-0,120, p<0,040).

Para determinar o grau de associação independente dos índices aterogênicos, realizou-se análise logística multivariada, ajustada para possíveis fatores confundidores (Tabela 4). Observou-se que os índices aterogênicos ΔIPP_90-120_ e o IAP foram preditores independentes de aterosclerose clínica.

**Tabela 4:** – Índices aterogênicos associados às doenças ateroscleróticas, conforme pontos de corte estabelecidos, segundo modelos de regressão logística

Índices aterogênicos	Modelo 1		Modelo 2		Modelo 3	

	OR (IC95%)	p	OR (IC95%)	p	OR (IC95%)	p
IC-I > 3,35	3,30 (1,92–5,67)	<0,001	1,85 (0,69–4,99)	0,224	-	-
ICL > 6,90	3,72 (1,90–7,28)	<0,001	1,05 (0,30–3,68)	0,933	-	-
IAP > 0,06	8,80 (4,93–15,73)	<0,001	4,06 (1,88–8,75)	<0,001	5,35 (2,30–12,45)	<0,001
ΔIPP_90-120_ ≤ 56,6	17,28 (9,49– 31,47)	<0,001	11,03 (5,58–21,80)	<0,001	9,58 (4,71–19,46)	<0,001

Modelo 1: sem ajuste; Modelo 2: ajustado por sexo, idade, história de tabagismo, IMC, prática regular de atividade física e presença de diabetes mellitus, hipertensão arterial e dislipidemias; Modelo 3: ajustado pelo modelo 2 + uso de estatina, pressão arterial diastólica e frequência cardíaca. IC-I: índice de Castelli I; ICL: índice de combinação de lipoproteínas; IAP: índice aterogênico plasmático; ΔIPP_90-120_: a variação do índice de perfusão periférica no intervalo 90-120 segundos após a desinsuflação do manguito.

## Discussão

Nosso estudo é o primeiro comparando novos índices aterogênicos em uma população brasileira. Os resultados presentes evidenciam uma importante associação independente entre a ΔIPP_90-120_ e o IAP com aterosclerose clínica. Consequentemente, o principal achado deste estudo diz respeito à possibilidade de utilização clínica de um derivado da oximetria de pulso e de relações derivadas da avaliação habitual de lipídeos. Outros estudos também encontraram associação independente do IAP com aterosclerose clínica^7,12-17,22^ e subclínica^4,10,19^ em diversos leitos arteriais.

Enquanto alguns estudos encontraram correlação inversa entre o IAP e a idade,^14,23^ um estudo em população africana concluiu que o IAP não estava associado à idade.^24^ Esta discrepância pode ser parcialmente o resultado das diferentes populações étnicas selecionadas. Em nosso estudo, de uma população brasileira majoritariamente de indivíduos não brancos (88,9%), foi encontrada correlação positiva com a idade e o IAP (r=0,173; p=0,003), o que pode ser explicado pela clássica associação entre a idade e o desenvolvimento de doenças ateroscleróticas.

Foi sugerido que valores de IAP de -0,3–0,1 estão associados a um baixo risco cardiovascular, 0,1–0,24 a médio risco e acima de 0,24 a alto risco.^25^ Condizente com os pontos de corte sugeridos, observamos IAP de 0,17 no grupo aterosclerose e -0,06 nos controles. Convém esclarecer que o uso elevado de estatinas no grupo aterosclerose pode justificar um IAP menor que o esperado,^25^ todavia, ele permaneceu significativamente maior neste grupo.

Verificou-se que o IAP estava negativamente associado ao diâmetro da partícula de LDL.^25^ Por consequência, um aumento no IAP indica uma redução no diâmetro da partícula de LDL e um aumento na proporção de partículas de LDL pequenas e densas (sdLDL).^25^Em situações de hipertrigliceridemia, há um estímulo à atividade da proteína de transferência de éster de colesterol (CETP), que está implicada na formação intravascular de sdLDL principalmente por meio de um mecanismo indireto envolvendo uma elevada taxa de transferência de ésteres de colesterol do HDL para partículas de VLDL1.^26-28^

Devido ao pequeno tamanho da partícula e também à ligação aumentada a proteoglicanos endoteliais, o sdLDL tem maior probabilidade de invadir e se depositar na parede arterial e de ser oxidado, o que leva a ainda mais aterosclerose.^29-31^ No entanto, devido às técnicas complexas e pouco custo-efetivas para a quantificação da fração sdLDL, sua aplicação na prática clínica costuma ser limitada, o que garante vantagem de custo ao IAP.

Neste estudo, a ΔIPP_90-120_ foi o preditor independente com maior associação ao desfecho. Há anos, evidências sugerem que a disfunção endotelial ocorre antes mesmo do processo de formação da placa aterosclerótica, contribuindo para sua formação, progressão e possíveis complicações.^32^ Menezes et al. sugeriram uma forma de avaliar a disfunção endotelial em indivíduos com aterosclerose clínica por meio da ΔIPP_90-120_, e seus resultados evidenciaram níveis reduzidos deste índice em indivíduos no grupo aterosclerose,^9^ independentemente do sexo, de forma bastante similar ao presente estudo. Ao ocorrer o estágio de disfunção endotelial, a resposta vasodilatadora está reduzida ou ausente, e a ΔIPP_90-120_ surge como uma possível ferramenta de avaliação desse estágio disfuncional no período em que ocorre maior contribuição do NO para os efeitos da hiperemia reativa.^9,33^

Apesar de não ter sido encontrada correlação entre o IAP e variáveis hemodinâmicas que pudessem justificar sua correlação encontrada com a ΔIPP_90-120_, alguns estudos relataram associação independente entre níveis plasmáticos de elevados de TG e reduzidos de HDL com a rigidez arterial.^34-36^

O processo que leva ao aumento da rigidez das grandes artérias é complexo e compreende influências mediadas por estresse mecânico pulsátil, fatores de crescimento e alterações na função endotelial, células inflamatórias, enzimas que degradam a elastina, alterações nas células musculares lisas do fenótipo contrátil para sintético e aumento da produção de matriz extracelular por fibroblastos.^37^ Sabe-se que os TG e o HDL têm influências opostas na inflamação, no estresse oxidativo, na formação da matriz extracelular e na alteração do músculo liso vascular do fenótipo contrátil para o sintético, e o IAP, de alguma forma, resume tais influências.^34,38^ Contudo, foram publicados achados contraditórios e, portanto, permanecem controvérsias sobre as associações do IAP com a rigidez arterial e, consequentemente, com a ΔIPP_90-120_.

Nosso estudo tem algumas limitações. A primeira delas ocorre pelo desenho observacional e transversal, realizado em centro único, que pode envolver um viés de seleção, limitando esta pesquisa apenas na geração de hipóteses. Em segundo lugar, nossos dados não puderam explicar completamente a relação fisiopatológica encontrada entre o IAP e a ΔIPP_90-120_. Outra limitação potencial é que foi realizada uma única medida dos índices avaliados para cada paciente, o que restringe as conclusões acerca da reprodutibilidade intra-individual dos métodos. Apesar dessas limitações, este estudo é o primeiro a comparar a relação dos novos índices aterogênicos em diversas patologias ateroscleróticas em uma população brasileira de pacientes ambulatoriais.

## Conclusão

Os resultados permitem concluir que o IAP e ΔIPP_90-120_ apresentaram melhor acurácia para discriminar aterosclerose clínica. Além disso, eles foram preditores independentes de aterosclerose clínica, evidenciando uma possibilidade promissora para o desenvolvimento de estratégias preventivas e de controle para doenças cardiovasculares. Tratam-se, portanto, de marcadores adequados para estudos multicêntricos do ponto de vista de praticidade, custo e validade externa.
