# Supplementary material for: Uso de Índices Aterogênicos como Métodos de Avaliação das Doenças Ateroscleróticas Clínicas
Source: Arq Bras Cardiol. 2023 Dec 15;120(12):e20230418. [Article in Portuguese] doi: 10.36660/abc.20230418 (PMC10789372; doi:10.36660/abc.20230418)
Supplement: Translation

# Translation: Use of Atherogenic Indices as Assessment Methods of Clinical Atherosclerotic Diseases

**Authors:** Yuri Barbosa Araújo, Ana Beatriz Rocha Almeida, Márcio Fellipe Menezes Viana, Rafael Alexandre Meneguz-Moreno

**Affiliations:** 1 Brazil Universidade Federal de Sergipe - Departamento de Medicina de Lagarto, Lagarto, SE – Brazil

**Keywords:** Atherosclerosis, Atherosclerotic Plaque, Lipoproteins, Perfusion Index

## Abstract

**Background:**

The search for clinically useful methods to assess atherosclerotic diseases (ASCVD) with good accuracy, low cost, non-invasiveness, and easy handling has been stimulated for years. Thus, the atherogenic indices evaluated in this study may fit this growing demand.

**Objectives:**

To assess the potential of atherogenic indices to evaluate patients with clinical atherosclerosis.

**Methods:**

Single-center cross-sectional study, through which the Castelli I and II indices, the atherogenic index of plasma (AIP), the lipoprotein combine index, and the variation in the peripheral perfusion index between 90 and 120 seconds after an endothelium-dependent (ΔPI_90-120_) vasodilator stimulus were evaluated in the prediction of atherosclerosis. Statistical significance was set at p < 0.05.

**Results:**

The sample consisted of 298 individuals with an average age of 63.0±16.1 years, of which 57.4% were women. Paired comparisons of the ROC curve analysis of the indices that reached the area under the curve (AUC) > 0.6 show that ΔPI_90-120_ and AIP were superior to other indices, and no differences were observed between them (difference between AUC = 0.056; 95%CI -0.003–0.115). Furthermore, both the ΔPI_90-120_ [odds ratio (OR) 9.58; 95%CI 4.71–19.46)] and AIP (OR 5.35; 95%CI 2.30–12.45) were independent predictors of clinical atherosclerosis.

**Conclusions:**

The AIP and ΔPI_90-120_ represented better accuracy in discriminating clinical ASCVD. Moreover, they were independent predictors of clinical ASCVD, evidencing a promising possibility for developing preventive and control strategies for cardiovascular diseases. Therefore, they are markers for multicenter studies from the point of view of practicality, low cost, and external validity.

## Introduction

Atherosclerosis is the central pillar of the pathophysiology of several cardiovascular diseases.^1^ Despite the widespread use of classic lipid parameters, widely available for clinical analysis, other parameters are currently being discussed, proposing associations of these lipid variables to assess their relationships and the correlation with clinical outcomes, especially coronary disease.^2-4^

In 1983, Castelli suggested the Castelli Indices I and II as a reflection of clearance of total cholesterol (TC) and LDL, both mediated by HDL levels.^5^ Recently, the atherogenic index of plasma (AIP) has gained scientific notoriety. It is speculated that the great predictive potential of AIP for atherosclerotic diseases derives from the ability of this index to indicate that the ratio between triglycerides (TG) and HDL can predetermine the preferential direction of intravascular transport of cholesterol towards beneficial HDL or atherogenic LDL.^6,7^ In the last 4 years, represented by the relationship between molar concentrations of TC, LDL, and TG with HDL, the lipoprotein combine index (LCI) was proposed as a possible independent predictor of coronary disease in menopausal women.^8^

Newly, a study showed encouraging results of the perfusion index (PI), a parameter derived from the pulse oximeter, in evaluating endothelial function in the presence of atherosclerosis. This same study reported that the interval of the PI variation between 90 and 120 seconds (ΔPI_90-120_) after reactive hyperemia seems to have the highest correlation between cardiovascular risk factors and endothelial dysfunction.^9^

Given the importance of endothelial dysfunction and lipid profile for developing and progressing atherosclerotic diseases, the search for useful clinical assessment methods with good accuracy, low cost, non-invasiveness, and easy handling has been stimulated for years. Considering the already described association of these indices with several cardiovascular clinical outcomes,^3,4,7,10-19^ they may fit the growing demand for cost-effectiveness and make them attractive for future trials and possible improvement in the detection, prevention, and treatment of such diseases. Thus, the present study aimed to evaluate the potential of atherogenic indices to predict clinical atherosclerotic disease.

## Methods

### Study design

This is an observational, cross-sectional study through which the values of the Castelli I and II indices, atherogenic index of plasma, lipoprotein combine index, and the variation of the peripheral perfusion index after an endothelium-dependent vasodilator stimulus were evaluated. Patients with clinical atherosclerosis in different vascular sites were included based on the common concomitance of sites involved and on their systemic characteristics.^20^

### Study location and sample

The present study was conducted in a cardiology, endocrinology, and geriatrics outpatient clinic linked to a tertiary hospital in northeastern Brazil. A retrospective sample size calculation was performed for the primary outcome with a 1:2 ratio for occurrence of the outcome, based on a previously conducted pilot study. With a power of 0.8, an α of 0.05, and an AUC=0.6 according to our a priori hypothesis (null hypothesis: AUC=0.5), a sample of 294 participants was required. To compensate for possible losses, 10% was added for sample adjustment, totaling 323 participants.

### Inclusion and exclusion criteria

All patients who attended the outpatient care of the specialties above would be invited to participate in the study provided they were at least 18 years old and had a lipidogram result collected up to 03 months before the inclusion in the research. Due to particular changes in lipid parameters, patients with familial hypercholesterolemia and users of protease inhibitors combined with oral contraceptives or isotretinoin were excluded from the study. Furthermore, since numerous factors can affect vascular reactivity, patients on dialysis, pregnant women, and patients who have exercised within 1 hour of the interview, or who have ingested energetic substances, or who have smoked at least 4 to 6 hours before the start of the interview from the data collect were also excluded from the study.

### Definition of clinical atherosclerotic disease

The patients had their diseases confirmed by the electronic medical record prepared by specialist physicians and complementary tests, including reports of coronary angiography and angiotomography of coronary arteries with atherosclerotic plaques with stenosis ≥ 50%, physical or pharmacological stress echocardiogram, pharmacological stress cardiac magnetic resonance, arteriography or arterial echo-doppler of the lower limbs and echo-doppler of the carotid arteries showing atherosclerotic plaques with stenosis ≥ 50%, in addition to tomography and angiotomography of the skull with signs of ischemia and cardioembolic etiologies excluded. Non-invasive tests were considered positive when ischemia was evidenced. Positive reports of atherosclerosis diagnosed within 1 year of the most recent lipidogram were considered.

### Allocation of groups

In this study, the clinical atherosclerotic disease (ASCVD) group comprised coronary artery disease, carotid or peripheral atherosclerotic disease, and atherothrombotic ischemic cerebrovascular disease. Thus, the control group consisted of those who did not have a diagnosed clinical atherosclerotic disease, individuals with subclinical atherosclerosis, or those without an atherosclerotic process.

### Data collection

Data was collected from January 2022 to December 2022 through interviews and physical examination in individualized rooms, with closed doors, respecting the participant’s privacy and the general data protection law. Respondents were randomly selected by active search on random days before outpatient care.

Variables related to cardiovascular risk were collected: gender, age, ethnicity, regular practice of physical activity, body mass index (BMI), dyslipidemia, type 2 diabetes mellitus, arterial hypertension, history of alcoholism, and current or previous smoking. Those who did not regularly practice at least 150 minutes of moderate physical activity were classified as inadequately practicing physical activity.^21^

The following atherogenic indices were calculated: Castelli I (CI-I) (CT/HDL) and Castelli II (CI-II) indices (LDL/HDL), the lipoprotein combine index (LCI) (CTxTGxLDL/HDL), the atherogenic index of plasma (AIP), calculated as log_10_(TG/HDL), and the variation in the peripheral perfusion index in the interval 90-120 seconds (ΔPI_90-120_) after cuff deflation, for AIP and LCI, lipid parameters (TC, LDL, HDL and TG) were expressed in mmol/L.

### PI collection

A portable pulse oximeter (model HC261, Multilaser, Brazil) was used for PI analysis. In this assessment, performed by a single investigator, patients were accommodated and seated for approximately 5 minutes in a silent room with a controlled temperature of 20-22ºC. The PI collect protocol followed the same used by Menezes et al.^9^ After cuff deflation, the PI value was evaluated and recorded at 90 and 120 seconds to evaluate its PI variation in this period (ΔPI_90-120_) using the following formula:


ΔPI:(PI time −PI baseline /PI baseline (x100)

### Statistical analysis

Variables with normal distribution were described as mean ± standard deviation, and variables without normal distribution were described as median and interquartile range. Continuous variables were evaluated using the Shapiro-Wilk analytical method to determine the normality of the distribution. The unpaired Student’s t-test was performed for variables with normal distribution and the Mann-Whitney U test for those without normal distribution. For categorical variables, Pearson’s chi-square test was used. Cutoff points for atherogenic indices were obtained using receiver operating characteristic (ROC) curves, chosen using the Youden index. The DeLong method calculated and compared areas under the curve (AUC). Furthermore, sensitivity, specificity, positive (PV+) and negative (PV-) predictive value, and positive (LR+) and negative (LR-) likelihood ratios for the outcome were recorded.

Pearson’s correlation analysis was performed to investigate the correlation of indices with the highest AUC with other continuous variables. To assess the degree of association between the variables and the outcome, odds ratios (OR) and their 95% confidence intervals (95%CI) were calculated for the presence of atherosclerotic disease using univariate logistic regression. Those that reached p<0.10 or that were considered clinically relevant were included in the multivariate model. P values <0.05 were considered statistically significant. Data were analyzed using SPSS, version 26.0 (SPSS Inc., Chicago, IL, USA) and MedCalc^®^, version 19.5 (MedCalc Software Ltd, Ostend, Belgium).

### Ethical aspects

This project was approved by the Research Ethics Committee, under opinion nº 5,106,513, according to the guidelines and norms established in resolution nº 466/2012 of the CNS, which deals with research with human beings.

## Results

During the research period, data from 323 volunteers were analyzed, of which 13 were excluded for contraceptives, 4 for using protease inhibitors, 5 for being dialytic patients, 2 for diagnosing familial hypercholesterolemia, and 1 for being pregnant. Thus, the final sample consisted of 298 participants (mean age 63 ± 16.1 years), of which 102 composed the clinical atherosclerosis group, while 196 participants without atherosclerosis or with subclinical atherosclerosis composed the control group. Among patients in the atherosclerosis group, the arterial beds most affected by clinical atherosclerosis were, respectively, the coronary (76), followed by the brain (26), carotid (9), and peripheral (4); 12 patients were diagnosed with more than one atherosclerotic disease. The baseline clinical characteristics of the studied groups are summarized in Table 1.

**Table 1:** – Comparison of the clinical characteristics of the studied population

	General population (n = 298)	Group		p

		Control (n = 196)	Atherosclerosis (n = 102)	
Age, (years)	63.0 ± 16.1	59.4 ± 17.0	70.0 ± 11.4	<0.001
Female, n (%)	171 (57.4)	123 (62.8)	48 (47.1)	0.009
Non-white, n (%)	265 (88.9)	172 (87.8)	93 (91.2)	0.372
BMI, (kg/m^2^)	28.4 ± 5.8	28.3 ± 6.4	28.6 ± 4.6	0.342
SBP, (mmHg)	132.6 ± 19.3	132.1 ± 18.1	133.5 ± 21.5	0.947
DBP, (mmHg)	80.1 ± 12.6	81.8 ± 12.0	77.0 ± 13.2	0.002
Heart rate (bpm)	75.5 ± 13.3	76.7 ± 12.8	73.3 ± 14.0	0.015
Type 2 diabetes mellitus, n (%)	128 (43.0)	70 (35.7)	58 (56.9)	<0.001
Dyslipidemias, n (%)	196 (65.8)	106 (54.1)	90 (88.2)	<0.001
Arterial Hypertension, n (%)	239 (80.2)	144 (73.5)	95 (93.1)	0.002
Smoking, n (%)	131 (44.0)	70 (35.7)	61 (59.8)	<0.001
Alcoholism, n (%)	106 (35.7)	64 (32.7)	42 (41.6)	0.128
Regular physical activity, n (%)	60 (20.1)	51 (26.0)	9 (8.8)	<0.001
ARB/ACE inhibitors, n (%)	212 (71.1)	131 (66.8)	81 (79.4)	0.023
Diuretics, n (%)	154 (51.7)	93 (47.4)	61 (59.8)	0.043
Beta-blockers, n (%)	117 (39.3)	50 (25.5)	67 (65.7)	<0.001
CCB, n (%)	83 (27.9)	50 (25.5)	33 (32.4)	0.211
Statins, n (%)	187 (62.8)	92 (46.9)	95 (93.1)	<0.001
Platelet antiaggregants, n (%)	104 (34.9)	19 (9.7)	85 (83.3)	<0.001

BMI: body mass index; SBP: systolic blood pressure; DBP: diastolic blood pressure; ACE: angiotensin-converting enzyme; ARB: angiotensin receptor blockers; CCB: calcium channel blockers.

Laboratory parameters and atherogenic indices are shown in Table 2. Among lipid parameters, differences were observed only between triglycerides and HDL levels. In the atherosclerosis group, higher levels of triglycerides and lower levels of HDL were observed. The atherogenic indices IC-I, IC-II, AIP, and ICL were significantly higher in the atherosclerosis group, whereas a lower median of ΔPI_90-120_ was observed.

**Table 2:** – Comparison of the laboratory parameters of the study groups

	General population (n = 298)	Group		p

		Control (n = 196)	Atherosclerosis (n = 102)	
Total cholesterol, (mg/dL)	175 (143 – 215)	178 (150 – 214)	162 (136 – 220)	0.250
Triglycerides, (mg/dL)	118 (85 – 158)	104 (77 – 138)	147 (114 – 188)	<0.001
HDL cholesterol, (mg/dL)	47 (39 – 56)	51 (42 – 59)	40 (34 – 47)	<0.001
LDL cholesterol, (mg/dL)	101 (72 – 132)	103 (74 – 134)	95 (69 – 132)	0.361
Non-HDL cholesterol, (mg/dL)	127 (95 – 162)	128 (93 – 160)	127 (99 – 168)	0.446
Hemoglobin, (g/dL)	13.2 ± 1.7	13.1 ± 1.7	13.2 ± 1.7	0.984
Hematocrit, (%)	39.9 ± 5.1	39.5 ± 5.0	40.6 ± 5.3	0.176
Platelets, (10 ^3^ /mL)	238 ± 72	236 ± 69	243 ± 75	0.902
Leukocytes, (10^3^/mL)	6.35 (5.20 – 7.8)	6.30 (4.98 – 7.70)	6.49 (5.50 – 7.85)	0.102
Urea, (mg/dL)	34 (27 – 44)	32 (26 - 40)	38 (29 - 50)	0.002
eGFR, (mL/min/1.73m^2^)	77 ± 23	81 ± 23	70 ± 22	<0.001
HbA1c, (%)	6.0 (5.5 - 6.8)	5.8 (5.4 - 6.4)	6.4 (5.9 - 7.7)	<0.001
Fasting blood glucose, (mg/dL)	97 (86 – 118)	93 (85 – 110)	105 (91 – 134)	<0.001
Castelli II Index	3.6 (3.0 - 4.7)	3.4 (2.8 - 4.3)	4.0 (3.4 - 5.4)	<0.001
Castelli II Index	2.1 (1.5 - 2.9)	2.0 (1.4 - 2.8)	2.3 (1.8 - 3.3)	0.011
Atherogenic index of plasma	0.03 (-0.12 – 0.20)	-0.06 (-0.20 - 0.11)	0.17 (0.08 - 0.36)	<0.001
Lipoprotein combine index	11.6 (6.7 - 24.2)	10.5 (5.6 - 20.3)	15.6 (8.4 - 35.7)	<0.001
Baseline PI, (%)	5.3 (3.1 - 8.0)	4.0 (2.7 - 6.1)	7.8 (5.5 - 9.8)	<0.001
ΔPI_90-120_, (%)	75.8 (45.3 - 130.3)	99.3 (69.4 - 157.8)	40.3 (8.1 - 65.0)	<0.001

HDL: high-density lipoprotein; LDL: low-density lipoprotein; eGFR: estimated glomerular filtration rate using the CKD-EPI formula; PI: peripheral perfusion index; ΔPI_90-120_: the variation in the peripheral perfusion index in the interval 90-120 seconds after cuff deflation.

Table 3 presents the sensitivity, specificity, predictive values, and likelihood ratios of the indices analyzed in this study. Note that only the Castelli II Index did not reach an AUC>0.6 (AUC=0.589). The ROC curves of these indices can be seen in Figure 1.

**Table 3:** – Sensitivity, specificity, predictive values, and likelihood ratios of atherogenic indices

Indices	Cutoff	AUC	SENS	SPEC	PV+	PV-	LR+	LR+
**IC-I**	> 3.35	0.658	77.5	49.5	44.4	80.8	1.53	0.46
IC-II	> 1.86	0.589	73.5	43.9	40.5	76.1	1.31	0.60
LCI	> 6.90	0.642	88.2	33.2	40.7	84.4	1.32	0.35
AIP	> 0.06	0.795	81.4	66.4	56.1	87.3	2.45	0.28
ΔPI _90-120_	≤ 56.6	0.851	72.5	86.7	74.0	85.9	5.47	0.32

AUC: area under the curve; SENS: sensitivity; SPEC: specificity; PV+: positive predictive value; PV-: negative predictive value; LR+: positive likelihood ratio; LR-: negative likelihood ratio; CI-I: Castelli I index; CI-II: Castelli II index; AIP: atherogenic index of plasma; LCI: lipoprotein combine index; ΔPI_90-120_: the variation in the peripheral perfusion index in the interval 90-120 seconds after cuff deflation.

**Figure 1:** – ROC curves atherogenic indices for atherosclerotic disease. CI-I: Castelli I index; CI-II: Castelli II index; LCI: lipoprotein combine index; AIP: atherogenic index of plasma; ΔPI90-120: the variation in the peripheral perfusion index in the interval 90-120 seconds after cuff deflation.

Paired comparisons of the ROC analysis of the indices that reached AUC > 0.6 show that, although there was no significant difference between the ΔPI _90-120_ and the AIP, both were shown to be greater than CI-I and LCI, between which no difference was observed either.

After observing the greater accuracy of the AIP and ΔPI_90-120_, Pearson’s correlation analysis was performed to investigate their correlations with other continuous variables. AIP was positively correlated with age (r=0.173, p=0.003), BMI (r=0.116, p=0.046), TC (r=0.138, p=0.017), TG (r=0.830, p<0.001), and was negatively correlated with HDL (r=-0.599, p<0.001) and ΔPI_90-120_ (r=-0.237, p<0.001). In turn, the ΔPI_90-120_ was positively correlated with DBP (r=0.154, p=0.012), HDL (r=0.321, p<0.001), and negatively correlated with age (r=-0.258, p<0.001), and TG (r=-0.120, p<0.040).

A multivariate logistic analysis was performed to determine the degree of independent association of the atherogenic indices, adjusted for possible confounding factors (Table 4). It was observed that the atherogenic indices ΔPI_90-120_ and AIP were independent predictors of clinical atherosclerosis.

**Table 4:** – Atherogenic indices associated with atherosclerotic diseases, according to established cutoff points, according to logistic regression models

Indices	Model 1		Model 2		Model 3	

	OR (95%CI)	p	OR (95%CI)	p	OR (95%CI)	p
CI-I > 3.35	3.30 (1.92–5.67)	<0.001	1.85 (0.69–4.99)	0.224	-	-
LCI > 6.90	3.72 (1.90–7.28)	<0.001	1.05 (0.30–3.68)	0.933	-	-
AIP > 0.06	8.80 (4.93–15.73)	<0.001	4.06 (1.88–8.75)	<0.001	5.35 (2.30–12.45)	<0.001
ΔPI _90-120_ ≤ 56.6	17.28 (9.49– 31.47)	<0.001	11.03 (5.58–21.80)	<0.001	9.58 (4.71–19.46)	<0.001

Model 1: without adjustment; Model 2: adjusted for sex, age, history of smoking, BMI, regular practice of physical activity, and presence of diabetes mellitus, arterial hypertension, and dyslipidemia; Model 3: adjusted by model 2 + statin use, diastolic blood pressure, and heart rate. CI-I: Castelli I index; LCI: lipoprotein combine index; AIP: atherogenic index of plasma; ΔPI_90-120_: the variation in the peripheral perfusion index in the interval 90-120 seconds after cuff deflation.

## Discussion

Our study is the first to compare new atherogenic indices in a Brazilian population. The present results show an important independent association between ΔPI_90-120_ and AIP with clinical atherosclerosis. Consequently, the main finding of this study concerns the possibility of clinical use of a derivative of pulse oximetry and relationships derived from the usual assessment of lipids. Other studies have also found an independent association between AIP and clinical^7,12-17,22^ and subclinical^4,10,19^ atherosclerosis in different vascular beds.

While some studies found an inverse correlation between the AIP and age,^14,23^ a study in an African population concluded that the AIP was not associated with age.^24^ This discrepancy may be partially the result of the different ethnic populations selected. In our study, of a Brazilian population mostly composed of non-white individuals (88.9%), a positive correlation was found between age and AIP (r=0.173; p=0.003), which the classic association between age and the development of atherosclerotic diseases can explain.

It has been suggested that AIP values of -0.3–0.1 are associated with low cardiovascular risk, 0.1–0.24 with intermediate risk, and above 0.24 with high risk.^25^ Consistent with the suggested cutoff points, we observed an AIP of 0.17 in the atherosclerosis group and -0.06 in the controls. It should be clarified that the high use of statins in the atherosclerosis group may justify a lower-than-expected AIP;^25^ however, it remained significantly higher in this group.

It was found that AIP was negatively associated with LDL particle diameter.^25^Consequently, an increase in the AIP indicates a reduction in the LDL particle diameter and an increase in the proportion of small dense LDL (sdLDL) particles.^25^ In situations of hypertriglyceridemia, there is a stimulus to the activity of cholesterol ester transfer protein (CETP), which is implicated in the intravascular formation of sdLDL mainly through an indirect mechanism involving a high rate of transfer of cholesterol esters from HDL to VLDL1 particles.^26-28^

Due to the small particle size and increased binding to endothelial proteoglycans, sdLDL is more likely to invade and deposit in the arterial wall and be oxidized, leading to even more atherosclerosis.^29-31^ However, due to the complex and low cost-effective techniques for quantifying the sdLDL fraction, its application in clinical practice is usually limited, guaranteeing a cost advantage to the AIP.

In this study, the ΔPI_90-120_ was the independent predictor with the greatest association with the outcome. For years, evidence has suggested that endothelial dysfunction occurs even before the process of atherosclerotic plaque formation, contributing to its formation, progression, and possible complications.^32^ Menezes et al. suggested a way to assess endothelial dysfunction in individuals with clinical atherosclerosis through the ΔPI_90-120_, and their results showed reduced levels of this index in individuals in the atherosclerosis group,^9^ regardless of gender in a way that is very similar to the present study. When the endothelial dysfunction stage occurs, the vasodilator response is reduced or absent, and the ΔPI_90-120_ appears as a possible tool for evaluating this dysfunctional stage in the period in which there is a greater contribution of NO to the effects of reactive hyperemia.^9,33^

Although no correlation was found between AIP and hemodynamic variables that could justify its correlation with ΔPI_90-120,_ some studies have reported an independent association between elevated plasma levels of TG and reduced HDL with arterial stiffness.^34-36^

The process leading to increased stiffness of large arteries is complex and comprises influences mediated by pulsatile mechanical stress, growth factors and changes in endothelial function, inflammatory cells, enzymes that degrade elastin, changes in smooth muscle cells from the contractile to synthetic phenotype, and increases extracellular matrix production by fibroblasts.^37^ It is known that TG and HDL have opposing influences on inflammation, oxidative stress, extracellular matrix formation, and the change of vascular smooth muscle from the contractile to the synthetic phenotype, and the AIP, somehow, summarizes these influences.^34,38^ However, contradictory findings have been published, and, therefore, controversies remain about the associations of AIP with arterial stiffness and, consequently, with ΔPI_90-120_.

Our study has some limitations. The first occurs due to the observational and cross-sectional design of the study, conducted in a single center, which may involve a selection bias, limiting this research only to the generation of hypotheses. Second, our data could not fully explain the pathophysiological relationship between AIP and ΔPI_90-120_. Another potential limitation is that a single measurement of the indices evaluated was performed for each patient, which restricts conclusions about the intra-individual reproducibility of the methods. Despite these limitations, this study is the first to compare the relationship of new atherogenic indices in different atherosclerotic pathologies in a Brazilian population of outpatients.

## Conclusion

The results allow us to conclude that the AIP and ΔPI_90-120_ presented better accuracy in discriminating clinical ASCVD. Furthermore, they were independent predictors of clinical ASCVD, evidencing a promising possibility for developing preventive and control strategies for cardiovascular diseases. Therefore, they are suitable markers for multicenter studies from the point of view of practicality, low cost, and external validity.
